# Outbreak of *Burkholderia cepacia *complex infections associated with contaminated octenidine mouthwash solution, Germany, August to September 2018

**DOI:** 10.2807/1560-7917.ES.2018.23.42.1800540

**Published:** 2018-10-18

**Authors:** Sören L. Becker, Fabian K. Berger, Susanne K. Feldner, Irem Karliova, Manfred Haber, Alexander Mellmann, Hans-Joachim Schäfers, Barbara Gärtner

**Affiliations:** 1 Institute of Medical Microbiology and Hygiene, Saarland University, Homburg/Saar, Germany; 2 Department of Thoracic and Cardiovascular Surgery, Saarland University, Homburg/Saar, Germany; 3 Hospital Pharmacy, Saarland University, Homburg/Saar, Germany; 4 Institute of Hygiene, University Hospital Münster, Münster, Germany

**Keywords:** *Burkholderia cepacia*, Hygiene, Infection prevention, Outbreak investigation, Whole-genome sequencing

## Abstract

Three German patients developed nosocomial pneumonia after cardiac surgery and had *Burkholderia cepacia *complex detected in respiratory specimens. Two patients died of septic multi-organ failure. Whole-genome sequencing detected genetically identical *B. cepacia *complex strains in patient samples, from a batch of octenidine mouthwash solution, which had been used for nursing care, as well as in samples obtained from the manufacturer during production. Contamination of medical products during manufacturing may lead to international outbreaks.

We report a nosocomial outbreak caused by *Burkholderia cepacia *complex (BCC) in a German cardiothoracic intensive care unit. Within 1 month, three critically ill patients were diagnosed with BCC in respiratory specimens. An outbreak investigation was launched, and whole-genome sequencing (WGS)-based typing identified an intrinsically contaminated octenidine mouthwash solution as the source of these infections.

## Description of cases

**Patient 1**. A male patient in his late 80s with arterial hypertension underwent repeat aortic valve replacement due to a paravalvular leak and cardiac decompensation. After surgery, he developed severe nosocomial pneumonia. Bronchial aspirates grew *Pseudomonas aeruginosa* and the empirical antibiotic treatment with meropenem was adjusted to ceftazidime. After moderate respiratory improvement, the patient’s condition deteriorated and he developed liver failure with jaundice and renal insufficiency. Twenty-three days after surgery, additional bronchial aspirates were sent for microbiological investigations and BCC was detected after 24 hours of incubation. Despite treatment with ceftazidime and tobramycin, the patient died from multi-organ failure.

**Patient 2**. A male patient in his late 70s underwent replacement of the ascending aorta and repeat aortic valve replacement due to valve degeneration and cardiac decompensation. *Morganella morganii* was found in several blood cultures and treated with piperacillin/tazobactam. On day 27 after surgery, BCC grew in respiratory specimens after 24 hours of incubation and the antibiotic treatment was changed to ceftazidime and tobramycin. The patient developed liver and renal failure, as well as haemodynamic instability necessitating vasopressor treatment. Despite escalation of the anti-infective treatment, the patient succumbed to septic multi-organ failure 9 days after detection of BCC.

**Figure 1:**
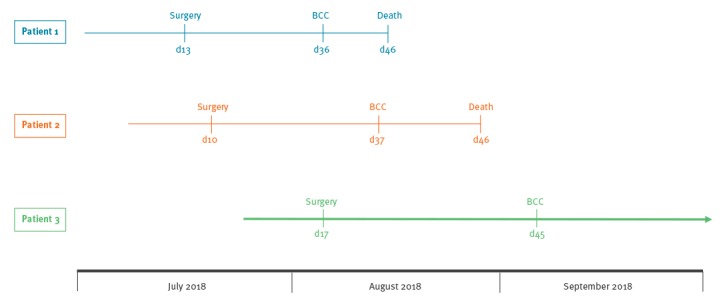
Timeline of three patients who underwent cardiac surgery and had *Burkholderia cepacia *complex (BCC) detected in respiratory specimens during inpatient treatment in a German University hospital, August–September 2018

BCC: *Burkholderia cepacia *complex; d: day number.

**Patient 3**. A male patient in his later 70s underwent infrarenal and bi-iliacal aortic replacement for an abdominal aortic aneurysm. Relaparotomy was required to manage a postoperative bleeding event. Nosocomial pneumonia due to *Enterobacter cloacae complex* and central line–associated infection caused by coagulase-negative staphylococci were successfully treated with meropenem and daptomycin, respectively. However, 28 days after surgery, the patient developed new pulmonary infiltrates; clinical signs of sepsis. *P. aeruginosa* and BCC were detected after 30 hours of incubation in bronchial aspirates (taken on day 28 after surgery) and the antibiotic regimen was adapted to ceftazidime. Due to respiratory insufficiency, the patient had to be intubated and a tracheostomy was required. His condition improved steadily and tracheostomy decannulation could be carried out on day 61 after surgery. As of 15 October 2018, the patient was still in hospital, but no longer requires treatment in an intensive care unit. The timeline between surgery and the detection of BCC of all three patients is displayed in Figure 1.

The coloured lines indicate the respective duration of inpatient treatment by number of days. The dates of surgery, the date of sampling of respiratory specimens that grew *Burkholderia cepacia complex *and the date of death for patients 1 and 2 are indicated.

## Outbreak investigation and environmental sampling

An outbreak investigation was launched following the unusual and rapid detection of BCC (after < 36 hours of incubation) in respiratory specimens from these patients. The first case was discussed in an infection prevention board together with neighbouring regional hospitals. No additional cases of BCC had been noted elsewhere, but one hospital reported having heard about an email sent by a manufacturer to the hospital’s pharmacy regarding a potential contamination with BCC in a mouthwash solution. Even though no such information had been received in the treating hospital, a targeted outbreak investigation was launched; samples were taken from octenidine mouthwash solution, other medical products that had been used for oral care of the affected patients and inhalers. Samples were tested to detect the presence of bacteria. BCC was detected in high quantity (> 1,000 colony-forming units/ml) in two unopened vials of the octenidine mouthwash batch that had been used during the 3 preceding weeks on the department’s normal care and intensive care unit (isolates O1 and O2). All BCC isolated showed similar colony morphology and identical antimicrobial susceptibility patterns, with relatively low minimal inhibitory concentrations (MICs) of ≤ 2 mg/L for ceftazidime, ceftolozan/tazobactam and co-trimoxazole.

**Figure 2:**
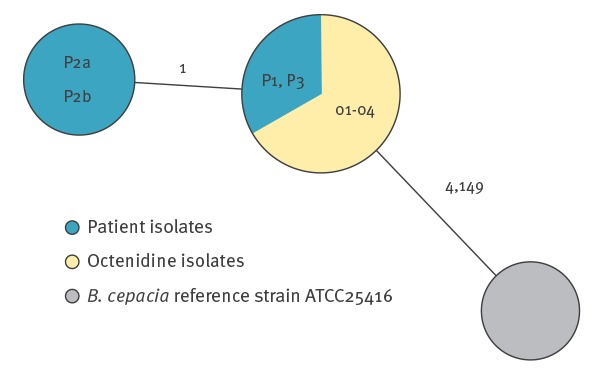
Minimum-spanning tree displaying the clonal relationship of the *Burkholderia cepacia *complex isolates detected during a hospital-based outbreak investigation in Germany, August–September 2018

We contacted the manufacturer and obtained two additional isolates of BCC, which were found in quality control examinations during the manufacturing process in summer 2018 (isolates O3 and O4). WGS-based typing of all isolates, comparing allelic profiles of all genes that could be extracted from the *de novo*-assembled genomes in comparison to reference strain ATCC 25416, resulted in identical or nearly identical genotypes among all suspected outbreak isolates (Figure 2). Raw reads of all isolates are available at [1] under the study accession number PRJEB28814.

O: octenidine isolate; P: patient isolate.

Each circle represents a genotype based on the allelic profile of 4,153 genes (Supplement) and is named by the according isolate(s). The diameter is proportional to the number of isolates included and coloured by the sample’s origin. The number on connecting lines between two circles displays the number of differing alleles in a pairwise comparison. For comparison, the allelic profile of reference strain ATCC 25416 (GenBank accession numbers NZ_CP012981-NZ_CP012983) is included.

## Discussion

BCC comprises a group of more than 20 closely related species of Gram-negative, non-fermentative bacteria. Although they are frequently found in water and fluid samples, diagnosis of BCC in the clinical microbiology laboratory can be challenging [2]. BCC is of considerable clinical relevance for patients with cystic fibrosis, who may develop difficult-to-treat pulmonary infections. It is intrinsically resistant to a wide range of antibiotics and antiseptics and has been shown to survive for at least 28 days in the presence of chlorhexidine gluconate and benzalkonium chloride [3]. There are several reports of pseudo-bacteraemia cases with BCC detected in blood cultures after application of contaminated chlorhexidine as skin antiseptic [4].

Given the ubiquitous occurrence of BCC in water, it is not surprising that several outbreaks related to contaminated medical devices or non-sterile products have occurred since the early 1990s. Most reports have been published in North America, e.g. a cluster of infections in a paediatric hospital linked to application of a contaminated nasal spray [5], infections on a neonatal intensive care unit that could be sourced to a contaminated blood gas analyser [6], and an ongoing outbreak over a 10-year-period associated with the use of contaminated ultrasound gel [7]. More recently, 17 infections were diagnosed in a hospital in Texas, United States (US) of America, which could be linked to manufacturer-contaminated vials of the laxative docusate [8]. A US-wide outbreak investigation was subsequently launched that identified a total of 108 cases in 12 federal states, all of which were sourced to contaminated water used in the production process of the liquid docusate [9]. Here, we illustrate the need for additional investigations beyond a single hospital or region whenever contamination during the manufacturing process is suspected.

Healthcare-associated infections due to BCC have also occurred in connection with contaminated Ringer-lactate infusions [10], mannitol solution [11], eye drops [12] and other drugs [13,14]. In Europe, recent outbreaks were reported from Spain [15], Switzerland [16] and Germany, the latter of which was associated with contaminated mouthwash solutions [17]. However, our report is the first to describe a contaminated octenidine-containing solution. The product is used as oral mouthwash as well as for decolonisation of methicillin-resistant *Stapylococcus aureus* (MRSA) and has thus direct contact with the mucous membranes in close proximity to a patient’s airways.

Here, we employed WGS, which is now widely accepted as the most accurate method to assess strain relatedness and to elucidate potential outbreaks [16]. Most previous reports on BCC outbreaks that were published before 2016 used alternative, less accurate methods for molecular typing, e.g. pulsed-field gel electrophoresis (PFGE), multilocus sequence typing (MLST) and repetitive palindromic PCR.

It remains unclear whether there was a causal association between the detection of BCC and the subsequent death of the first two patients who had considerable comorbidities (e.g. liver failure and renal insufficiency) and were infected with other pathogens (e.g. *P. aeruginosa*). Moreover, it cannot be fully excluded that the second of the three cases was due to BCC transmission on the ward; this was deemed unlikely, however, based on the results obtained through microbiological analysis of the octenidine mouthwash. Additionally, further patients colonised with MRSA or multiresistant Gram-negative pathogens were also treated on the same ward during that period and no cases of nosocomial transmission were observed, which suggests adherence to infection prevention measures. Some additional cases of BCC infection may not have been detected due to the difficult microbiological diagnosis of this bacterium, which frequently requires prolonged incubation for more than 48 hours and the use of several agar media. However, all departments in our hospital, in which octenidine mouthwash solution had been used, were informed about the potential exposure of their patients and prolonged microbiological incubation was applied to all respiratory samples from the affected wards.

Similar to the 2016 outbreak associated with contaminated docusate in the US [8,9], parts of the contaminated octenidine mouthwash batch have been dispatched to other hospitals in Germany and neighbouring European countries. It is possible, therefore, that additional cases in other locations/countries might have gone unnoticed. The manufacturer voluntarily launched a product recall in August 2018 and sent the product recall document by email to pharmacies, doctors, clinics, care homes, distributors and warehouses, but it is unknown whether all concerned healthcare institutions received the information. Hence, we have informed the respective regional and national public health authorities, which have launched an in-depth investigation at both national and international levels to elucidate the extent of the current outbreak and to detect additional cases in other hospitals.

## Conclusion

Intrinsic contamination of medical devices, antiseptics or drugs with BCC during production may give rise to severe, potentially fatal infections. Detection of this bacterium outside typical at-risk patients should always prompt investigations to exclude nosocomial sources and to prevent healthcare-associated outbreaks. In this study, active communication with the manufacturer and targeted information about the possible source of contamination allowed for a fast outbreak investigation. Notification of such events to public health authorities and rapid dissemination of outbreak-related information is crucial to detect further cases. There is an urgent need for regulations pertaining to the communication policy between manufacturer and end-users in case of a suspected contamination as well as microbiological surveillance and quality control during manufacturing of non-sterile medical products.
